# Health-related quality of life in syndromic scoliosis treated by segmental pedicle screw instrumentation: a case-control study

**DOI:** 10.2340/17453674.2025.44760

**Published:** 2025-11-17

**Authors:** Henri SALLINEN, Antti SAARINEN, Arimatias RAITIO, Matti AHONEN, Ilkka HELENIUS

**Affiliations:** 1 Department of Orthopedics and Traumatology, University of Turku and Turku University Hospital, Turku; 2 Department of Paediatric Surgery, Orthopaedics and Traumatology, University of Turku and Turku University Hospital, Turku; 3 Department of Paediatric Surgery and Orthopaedics, University of Helsinki and Helsinki University Hospital, Helsinki; 4 Department of Orthopedics and Traumatology, University of Helsinki and Helsinki University Hospital, Helsinki, Finland

## Abstract

**Background and purpose:**

Deformity correction with pedicle screw instrumentation in adolescent idiopathic scoliosis has been shown to improve the health-related quality of life (HRQoL), while evidence on syndromic scoliosis is limited. Syndromic patients tend to have comorbidities resulting in a higher risk of perioperative complications. We aimed to assess the change in HRQoL in patients with syndromic scoliosis compared with adolescent idiopathic scoliosis after spinal fusion with pedicle screw instrumentation.

**Methods:**

We conducted a retrospective case-control study in children undergoing segmental pedicle screw instrumentation for syndromic scoliosis between 2009 and 2023 with a 2-year follow-up. For each syndromic patient, 2 controls with adolescent idiopathic scoliosis were matched for sex and age. The Scoliosis Research Society-24 (SRS-24) questionnaire was used to assess HRQoL preoperatively and at follow-up.

**Results:**

35 syndromic (mean age 14.1 years) and 70 adolescent idiopathic patients (mean age 15.1 years) were included. The SRS-24 total score remained stable from preoperative to 2-year follow-up in both groups (median difference for change 0.05, 95% confidence interval [CI] –0.40 to 0.30). The pain domain improved 0.9 points (standard deviation [SD] 0.9) in syndromic and 1.0 points (SD 0.7) in adolescent idiopathic scoliosis patients. Non-ambulatory patients had greater improvement in pain than ambulatory syndromic patients (mean difference between groups 0.66, CI 0.15–1.24). 8 syndromic patients had complications compared with 3 adolescent idiopathic scoliosis patients (risk ratio 5.3, CI 1.5–19). One syndromic patient died during follow-up.

**Conclusion:**

The health-related quality of life in patients with syndromic scoliosis was comparable to patients with adolescent idiopathic scoliosis after posterior spinal fusion with segmental pedicle screw instrumentation.

Scoliosis in adolescents may be classified according to the etiology of the deformity as idiopathic (AIS), congenital, neuromuscular, or syndromic scoliosis (SS) [1,2]. SS is associated with an underlying systemic disease, such as Marfan syndrome, neurofibromatosis, arthrogryposis, and Down syndrome. In these patients, the incidence of scoliosis is significantly higher than in the general population [1]. SS includes a wide range of different conditions that can cause hypermobility, muscle pathology and poor bone quality. often leading to large, progressive curvatures that can cause respiratory problems, disturbed sitting balance, and back pain. SS typically responds poorly to bracing and therefore operative treatment is often needed [2].

Patients with SS tend to have comorbidities, which are associated with a high risk of perioperative complications [1,3]. Mechanical complications are more prevalent in SS, as patients typically present with reduced bone quality, small bony structures, and short and angular deformities with limited bone formation [2].

In patients with AIS, the association of pedicle screw instrumentation and health-related quality of life (HRQoL) has been studied comprehensively, while data for SS is limited. Previous studies indicate that instrumented spinal fusion improves long-term HRQoL more than observation in AIS [4,5]. As SS patients have a higher risk of complications, it is important to assess the impact of spinal surgery on the HRQoL, not only on radiographic parameters.

Our aim was to compare the change in HRQoL after spinal fusion in SS and AIS. Our secondary aim was to evaluate the incidence of complications between groups and predictors of HRQoL.

We hypothesized that SS patients undergoing spinal fusion would have more complications than AIS patients. Additionally, their change in HRQoL might be inferior to that of AIS patients.

## Methods

### Study design

Our study was a single-center case-control study from a tertiary level hospital with prospective data collection from an institutional pediatric spine register.

This study is reported following the Strengthening the Reporting of Observational Studies in Epidemiology (STROBE) guideline.

### Population

Children undergoing segmental pedicle screw instrumentation for SS between 2009 and 2023 with a standardized perioperative protocol comparing SS and AIS were included. For each SS patient, 2 control patients with AIS operated on at the same institution were matched manually for sex and age (± 3.5 years) at time of operation. A non-responder analysis was done for patient characteristics and total Scoliosis Research Society-24 (SRS-24) score (Table 1, see Appendix).

Perioperative management and follow-up were standardized. All patients had upright spinal radiographs preoperatively and at 2-year follow-up. Standing radiographs were obtained, if possible (25 SS patients, and all AIS patients); sitting radiographs were taken of non-ambulatory SS patients (n = 10). SS patients with known syndromes associated with neural axis abnormalities, such as neurofibromatosis type 1 or Marfan syndrome, and all AIS patients underwent spinal MRI preoperatively.

### Surgery

Surgical planning was done preoperatively, including types of implants, the need for posterior column osteotomies, and fusion levels. The need for posterior column osteotomy was evaluated case by case. Osteotomies were performed if adequate curve correction was not achieved without. 8 patients had kyphoscoliosis (T5–T12 kyphosis > 40°), which indicated posterior column osteotomies. In syndromic scoliosis, fusion levels were chosen based on ambulatory status. Ambulatory SS patients with no major pelvic obliquity (< 10°) were fused to the first stable vertebra. Non-ambulatory SS patients had pelvic fixation. In the AIS group, fusion level was determined by the Lenke classification [6]. For Lenke 1 and 2 curves the last substantially touched vertebra was used as the lowest instrumented level, and for Lenke 3–6 curves spinal fusion was continued to L3 or L4 [7].

Posterior elements were exposed using electrocautery. Patients with pelvic fusion typically received allograft, while patients with shorter fusion received local autograft from facetectomies or posterior column osteotomies with bone graft extenders. Both groups underwent segmental, bilateral pedicle screw instrumentation aiming for 2.0 screw density per instrumented vertebra. Corrective maneuvers were performed to obtain a well-corrected spinal deformity in both the coronal and sagittal plane, aiming for > 70% scoliosis correction in both groups. All operations were done by a single experienced pediatric orthopedic spine surgeon with an attending pediatric orthopedic surgeon. Bilateral segmental pedicle screw instrumentation (6.35 Legacy, Solera 6.0, Medtronic Spinal and Biologics, Minneapolis, MN, USA; Mesa2, Stryker Spine, Kalamazoo, MI, USA) was used for spinal deformity correction. CD Legacy was used for 12 SS patients and 21 AIS patients. Solera was used for 19 SS patients and 28 AIS patients. Mesa2 was used for 4 SS patients and 21 AIS patients. 20 SS patients received morselized femoral allograft. 15 SS patients and all AIS patients received bone graft extenders (tricalcium phosphate, Nanostim Medtronics, Minneapolis, MN, USA; iFactor, CeraPedics, Westminster, CO, USA). A single closed-suction subfascial drain was used for all patients for the first 24 hours postoperatively.

All patients had intraoperative neurophysiological monitoring. Lumbar nerve root electromyography, motor evoked potentials, and somatosensory evoked potentials were done at specific timepoints: incision, exposure, pedicle screw insertion, correction complete, and wound closure.

### Outcomes

Our primary outcome measure was the difference in SRS-24 domains between the groups from preoperative to 2-year follow-up. To measure the health-related quality of life, we used a Finnish unvalidated version of the SRS-24 questionnaire. SRS-24 contains 24 questions, which are scaled from 1 to 5. The score for each domain and the total score are divided by the number of questions in that domain. The minimum score for each domain is 1 and maximum is 5. The same applies to the total score. Higher scores mean better outcomes. SRS-24 domains are divided into 7 categories: pain, general and postoperative function, general and postoperative self-image, general activity level, and satisfaction [8].

The questionnaire was filled in preoperatively and at 24 months after surgery by the patient or the caregiver depending on the patient’s age and ability. Preoperatively only the first 15 questions were answered. The preoperative questionnaire was available for all SS patients. Postoperatively SRS-24 was available for 34/35 SS patients as 1 patient died before the 2-year follow-up. All AIS patients had both questionnaires available.

Secondary outcomes included perioperative characteristics, radiographic correction, and complication rates.

Scoliosis correction was measured from radiographs using the Cobb angle with the following formula: (preoperative Cobb angle–postoperative Cobb angle)/preoperative Cobb angle.

Complication was defined as any adverse event during or after surgery, such as dural tears, cerebrospinal fluid leak, massive blood loss (> 50% of blood volume), loss of motor evoked potential intraoperatively, new neurologic deficit, postoperative superficial or deep surgical site infection, breakage of instrumentation during follow-up, or any reoperation after index fusion surgery.

There was no missing data for these outcomes in any group.

### Statistics

All statistical analyses were performed using R version 4.5.1 (R Foundation for Statistical Computing, Vienna, Austria). Continuous variables were assessed for normality using the Shapiro–Wilk test and analyzed using nonparametric bootstrapping to estimate between-group differences with 95% confidence intervals (CI). 3 variables (age, major curve at 2-year follow-up, and change in pain domain) were of normal distribution and all other variables were of non-normal distribution. Summary statistics were reported as mean and standard deviation (SD) or median and interquartile range (IQR). Subgroup comparisons were performed between 3 groups: AIS, ambulatory syndromic scoliosis, and non-ambulatory syndromic scoliosis.

Categorical variables were analyzed using contingency tables, and risk ratios (RR) with CIs were calculated for the risk of complications. Differences in total number of complications were analyzed using Poisson regression and no over-dispersion was detected in the model diagnostics.

SRS-24 scores and their domains were analyzed using bootstrapped pairwise comparisons. Demographic factors potentially associated with postoperative health-related quality of life (HRQoL) were examined using bivariate analyses.

### Ethics, data sharing, funding, and disclosures

Ethical committee approval was obtained for our study (ETMK 96/1801/2020). All the patients and their parents provided written consent preoperatively. The data supporting these findings is available upon request. Personal research funds were received by the following authors: HS received grants from Clinical Research Institute HUCH. AS has received research grants from Clinical Research Institute HUCH, Päivikki and Sakari Sohlberg Foundation, and Vappu Uuspää Foundation. AR has received research grants from Päivikki and Sakari Sohlberg Foundation and Clinical Research Institute HUCH. MA has received research grants from Pediatric Research Foundation Finland and Nordforsk. IH has received research grants from Finnish Paediatric Research Foundation, Liv och Hälsa, Päivikki and Sakari Sohlberg Foundation, and Finnish State Funding via Helsinki and Turku University Hospitals. All authors report no conflicts of interest. Complete disclosure of interest forms according to ICMJE are available on the article page, doi: 10.2340/17453674.2025.44760

## Results

59 SS patients underwent segmental pedicle screw instrumentation (Figure). Of these patients, 24 were excluded (1 patient was too young to match, 17 patients failed to answer the preoperative questionnaire, and 6 patients declined to answer at follow-up). 1 SS patient died within 30 days of surgery. This patient was included without the follow-up questionnaire answered as death represents a serious adverse event and outcome of surgery. Of the remaining 35 patients, 30 underwent definitive fusion as primary treatment and 5 patients had growth-friendly treatment before definitive fusion. Thus, 35 children with SS and 70 with AIS were included. The mean age was 14.1 years (SD 2.6) for SS patients, and 15.1 years (SD 2.0) for AIS patients (Table 2). The most common syndromes in the SS group were neurofibromatosis type 1 (n = 6), arthrogryposis (n = 3), and Down syndrome (n = 3) (Table 3).

**Figure:**
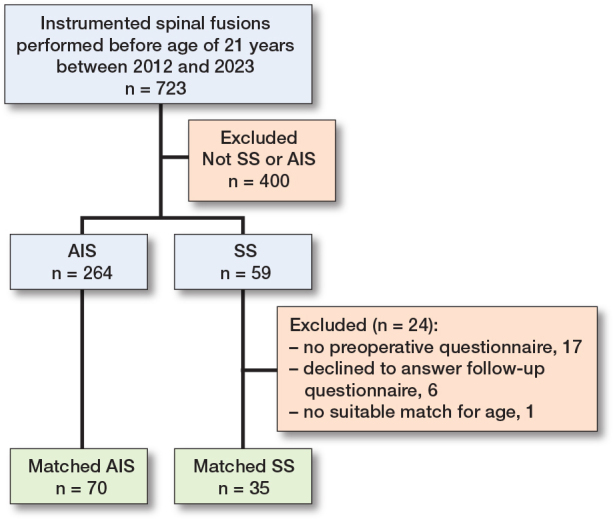
Flowchart of the patient cohorts.

**Table 2:** Clinical characteristics of the study groups

Characteristic	Syndromic scoliosis (n = 35)	Adolescent idiopathic scoliosis (n = 70)	Difference between groups (CI)
Age at surgery, years ^a^	14.1 (2.6)	15.1 (2.0)	1.0 (–0.1 to 1.9)
Female sex, n (%)	24 (69)	48 (69)	
Major curve, Cobb angle (°)			
Preoperative ^b^	54° [14]	51° [10]	3.0 (–4.0 to 6.0)
Postoperative ^b^	15° [8.5]	12° [6]	3.0 (–1.5 to 4.0)
Correction,% ^b^	70 [20]	70 [10]	0.02 (–0.04 to 0.1)
2-year follow up ^a^	18° (8)	14° (6)	3.2 (0.3 to 6.4)
Blood loss, mL ^b^	700 [438]	420 [446]	280 (10 to 400)
Operative time, hours ^b^	3.7 [1.1]	2.9 [1.2]	0.8 (0.3 to 1.2)
Number of levels fused ^b^	14 [5]	11 [2]	3 (1 to 4)
Iliac screws/S2AI screws, n	4/6	0/0	
Blood loss/time, mL/h ^b^	169 [99]	153 [85]	16 (–7 to 72)
Blood loss/level, mL/vertebra ^b^	44 [32]	40 [33]	4 (–6 to 17)

a Mean (SD). Difference between groups is mean (CI).

b Median [IQR]. Difference between groups is median (CI).

CI = 95% confidence interval.

**Table 3:** List of syndromes in patients with syndromic scoliosis (total = 35 patients)

Diagnosis	Number of patients
Neurofibromatosis type 1	6
Arthrogryposis	3
Down syndrome	3
Charcot-Marie-Tooth	2
Chromosome 15 long arm deletion	2
Coffin–Lowry syndrome	2
Osteogenesis imperfecta	2
Prader–Willi syndrome	2
Rett’s syndrome	2
Unknown syndrome	2
Angelman syndrome	1
CATCH-22	1
Cartilage-hair hypoplasia	1
Chromosome translocation	1
Marfan’s syndrome	1
Mitochondrial encephalopathy	1
Pterygium syndrome	1
Ehlers–Danlos syndrome	1
Sturge–Weber syndrome	1

### Clinical characteristics

The number of levels fused was greater in SS (median difference 3 levels, CI 1–4). 10 SS patients required pelvic fixation (29%) whereas none of the AIS patient had pelvic fixation. There were no significant differences in major curves preoperatively or postoperatively. At 2-year follow-up SS patients had larger residual curves (mean difference 3.2°, CI 0.3–6.4). Curve correction was 70% at 2-year follow-up for both groups (see Table 2).

Operative time was longer (median difference 0.8 hours, CI 0.3–1.2) and intraoperative blood loss was larger in the SS group (median difference 280 mL, CI 10–400). Intraoperative blood loss adjusted to operative time (mL/hours) or fused vertebrae (mL/vertebra) showed no significant differences between groups (see Table 2).

Comparison of patient characteristics between non-ambulatory and ambulatory SS patients is provided in Table 4 (see Appendix).

### SRS-24

There was no significant difference between SS and AIS preoperatively in the SRS-24 total score or any of its domains. The SRS-24 total score remained stable at the 2-year follow-up in both groups. The SRS-24 pain domain improved 0.9 points mean change (SD 0.9) in SS and 1.0 points mean change (SD 0.7) in AIS. At 2-year follow-up no difference was evident in any of the domains or the total score (Table 5).

**Table 5:** SRS-24 outcome questionnaire preoperatively and at 2-year follow-up

SRS-24	Syndromic scoliosis (n = 35)	Adolescent idiopathic scoliosis (n = 70)	Difference between groups (CI)
Total score			
Preoperative ^b^	4.1 [0.7]	4.1 [0.8]	0.1 (–0.4 to 0.3)
2-year follow up ^b^	4.1 [0.4]	4.1 [0.6]	0.04 (–0.2 to 0.2)
Change ^b^	–0.1 [1.1]	–0.1 [0.7]	0.05 (–0.4 to 0.3)
Pain			
Preoperative ^b^	3.6 [0.8]	3.4 [1.0]	0.1 (–0.3 to 0.4)
2-year follow up ^b^	4.6 [0.5]	4.6 [0.7]	0.0 (–0.1 to 0.4)
Change ^a^	0.9 (0.9)	1.0 (0.7)	0.1 (–0.2 to 0.5)
General self-image			
Preoperative ^b^	4.0 [1.5]	3.7 [1.0]	0.3 (–0.3 to 0.7)
2-year follow up ^b^	4.3 [1.0]	4.2 [1.0]	0.2 (–0.5 to 0.7)
Change ^b^	0.3 [1.3]	0.3 [1.3]	0.0 (–0.5 to 0.5)
Function for back condition			
Preoperative ^b^	4.0 [1.0]	4.3 [0.3]	0.3 (–0.2 to 0.7)
2-year follow up ^b^	4.3 [0.7]	4.3 [0.3]	0.0 (–0.3 to 0.0)
Change ^b^	0.0 [0.9]	0.0 [0.3]	0.0 (0.0 to 0.3)
General activity			
Preoperative ^b^	4.7 [1.5]	5.0 [0.7]	0.3 (–0.3 to 1.3)
2-year follow up ^b^	4.5 [0.9]	5.0 [0.3]	0.5 (0.0 to 0.7)
Change ^b^	0.0 [0.6]	0.0 [0.6]	0.0 (0.0 to 0.0)
Postoperative			
self-image ^b^	3.0 [0.9]	3.0 [0.7]	0.0 (0.0 to 0.7)
function ^b^	3.0 [1.0]	3.0 [1.0]	0.0 (0.0 to 0.0)
satisfaction ^b^	4.7 [0.9]	4.3 [0.7]	0.3 (–0.3 to 0.5)

a and b See Table 2.

### Association of ambulatory status with SRS-24

10 (29%) SS patients were non-ambulatory preoperatively and the fusion was extended to the pelvis. When comparing SS patients based on their ambulatory status, non-ambulatory SS patients had lower scores for general activity both preoperatively (median difference 1.0 points, CI 0.3–2.2) and at follow-up (0.7 points, CI 0.3–1.0). Non-ambulatory SS patients had greater improvement in pain domain (mean change 1.4 points, SD 0.9 vs 0.7 points, SD 0.9 with mean difference 0.7 points, CI 0.2–1.2). Change in pain domain for non-ambulatory patients was better also compared with AIS (mean difference 0.4 points, CI 0.01–0.8). There were no significant differences in other domains or total score (Table 6).

**Table 6:** SRS-24 outcome questionnaire preoperatively and at 2-year follow-up for SS patients based on ambulatory status

SRS-24	Non-ambulatory n = 10	Ambulatory n = 25	Difference between groups (CI)
Total score			
Preoperative ^b^	3.7 [1.1]	4.2 [0.7]	0.5 (–0.3 to 1.2)
2-year follow up ^b^	3.9 [0.4]	4.1 [0.5]	0.2 (–0.2 to 0.4)
Change ^b^	0.4 [1.0]	–0.2 [0.8]	0.6 (–0.1 to 1.2)
Pain			
Preoperative ^b^	3.4 [1.1]	3.7 [0.6]	0.4 (–0.6 to 1.1)
2-year follow up ^b^	4.4 [0.3]	4.6 [0.6]	0.1 (–0.3 to 0.3)
Change ^a^	1.4 (0.6)	0.7 (0.9)	0.7 (0.2 to 1.2)
General self-image			
Preoperative ^b^	4.3 [1.7]	4.0 [1.3]	0.3 (–1.3 to 1.0)
2-year follow up ^b^	4.3 [1.3]	4.3 [1.0]	0.0 (–1.0 to 1.0)
Change ^b^	0.3 [0.7]	0.3 [1.3]	0.0 (–1.0 to 1.0)
Function for back condition			
Preoperative ^b^	3.7 [1.2]	4.3 [0.7]	0.7 (–0.3 to 1.3)
2-year follow up ^b^	4.0 [0.7]	4.3 [0.3]	0.3 (–0.3 to 0.7)
Change ^b^	0.3 [1.0]	0.0 [0.7]	0.3 (0.0 to 1.3)
General activity			
Preoperative ^b^	3.7 [1.2]	4.7 [0.7]	1.0 (0.3 to 2.2)
2-year follow up ^b^	4.0 [0.7]	4.7 [0.7]	0.7 (0.3 to 1.0)
Change ^b^	0.3 [1.3]	0.0 [0.3]	0.3 (0.0 to 2.0)
Postoperative			
self-image ^b^	3.0 [0.7]	3.0 [1.0]	0.0 (–0.6 to 0.7)
function ^b^	3.0 [1.0]	3.0 [1.0]	0.00 (0.00 to 2.00)
satisfaction ^b^	4.3 [0.7]	4.7 [0.7]	0.33 (–0.33 to 0.67)

a and b See Table 2.

### Predictors of postoperative SRS-24 in syndromic scoliosis

Neither age, sex, nor residual major curve was associated with the postoperative SRS-24 total score or separate domains.

Longer fusions were associated with worse general activity scores (correlation coefficient [r] –0.46, CI –0.75 to –0.29) at follow-up. Larger blood loss during surgery was associated with worse general self-image (r –0.35, CI –0.59 to –0.06). Having a complication had a negative impact on the pain domain score at follow-up (r –0.23, CI –0.40 to –0.03). Complications had no association with any other domain, or the SRS-24 total score.

### Complications

The total number of complications was 10 for SS and 3 for AIS group (RR 6.7, CI 2.0–30). 8 patients in the SS group had at least 1 complication when compared with 3 patients in the AIS group (RR 5.3, CI 1.5–19). None of the patients had unplanned revision. One SS patient died within 30 days of the operation. The cause of death was pulmonary aspiration. Non-ambulatory SS patients had 4 complications, and ambulatory SS patients 6. Allograft use was not associated with deep surgical site infection or non-union. Dural tears or cerebrospinal fluid leaks were not observed in patients undergoing posterior column osteotomies. A complete list of complications and outcomes is given in Table 7.

**Table 7:** Intra- and postoperative complications during 2-year follow-up

Patient number Diagnosis		Complication	Outcome
4	Chromosome 15 deletion	Broken rod at 2-year follow-up	Asymptomatic, mild positive sagittal balance, follow-up
8	CATCH-22	Death within 30 days of operation	Cause of death: aspiration leading to cardiac arrest
11	Chromosomal anomaly	Broken S2AI screw at 6 months, broken rod at 2 years	Asymptomatic, follow-up
13	Rett’s syndrome	Intraoperative motor evoked potential change	Intraoperative screw revision. No clinical sequalae
23	Down syndrome	Broken rod at 2 years	Asymptomatic, follow-up
29	Pterygium syndrome	Cerebrospinal fluid leak	Full recovery
31	Open etiology syndrome	Cerebrospinal fluid leak	Full recovery
32	Neurofibromatosis type 1	Motor evoked potential change, pneumothorax caused by a screw	Chest tube, no neurologic deficit
49	AIS	Motor evoked potential change during correction	Responded to intraoperative elevated mean arterial pressure, no neural deficits
54	AIS	Superficial surgical site infection.	Antibiotics, full recovery
56	AIS	Misplacement of pedicle screw, screw removed intraoperatively	No neural deficit

## Discussion

We aimed to assess the impact of spinal fusion on HRQoL in syndromic scoliosis as compared with adolescent idiopathic scoliosis. We showed no change in the total SRS-24 score in either of the groups and no significant difference was evident in the change for other domains. Non-ambulatory SS patients had worse general activity scores both preoperatively and at follow-up but had greater improvement in the pain domain. Regarding complications, SS patients had more and 1 died within 30 days of operation.

### Comparison with previous data

Hsu et al. [9] reported that patients with SS had lower HRQoL scores in physical and multiple psychosocial domains than healthy controls. However, they did not report the impact of spinal fusion surgery on HRQoL.

Patients with neuromuscular scoliosis (NMS) have been shown to benefit from spinal fusion surgery regarding HRQoL. Soini et al. found that improvement was evident in multiple HRQoL domains for NMS [10]. Ersberg and Gerdhem reported improvement in general HRQoL at 2-year follow-up [11]. As many non-ambulatory SS patients share similar characteristics to NMS, it can be assumed that non-ambulatory SS patients also benefit from spinal fusion, which is supported by our finding that non-ambulatory patients had greater improvement in the pain domain than ambulatory groups.

Unfortunately, there are no established levels for minimum clinically significant differences (MCID) for SRS-24. For SRS-22r there are MCIDs for pain, activity, and appearance [12]. The MCID for pain domain in the SRS-22r has been established at 0.20 points. Conversion from SRS-24 to SRS-22r has been studied and conversion for pain domain has a good level of accuracy [13]. In our study, the SS group had improvement of 0.9 points in the pain domain.

Chung et al. reported major complications in 2.7% SS and in 1.0% AIS, and minor complications in 41% vs 28.5% [3]. Similarly, we showed that the risk of complications was significantly higher in SS. Deveza et al. reported comparable fusion length with the mean number of levels fused being 13.8 [14]. Reames et al. reported a 2.3 times higher risk of complications in SS than in AIS in the Scoliosis Research Society Morbidity and Mortality database [15].

Greater blood loss in syndromic scoliosis has been reported by Levy et al. regardless of the syndrome or surgical approach with average blood loss ranging from 903 to 2,106 mL between syndromes [1]. In our data, however, the blood loss was dependent on the operative time and number of fused levels, not the diagnosis itself. Jain et al. [16] reported that normalized blood loss (mL/kg/level fused) was greater in SS (2.46) than in AIS (1.52) [16].

### Strengths

The strengths of this study include prospective data collection and standardized perioperative management and follow-up for both groups.

### Limitations

Use of the SRS-24 questionnaire is not ideal for SS patients as it was developed for evaluating HRQoL in AIS [8]. However, the use of similar questionnaires for both groups allowed direct comparison.

The Finnish version of SRS-24 has not been validated for SS or AIS. The use of similar estimates of HRQoL in patients with AIS and SS made comparative study possible. The choice of questionnaire was done before our registry started in 2009 and SRS-24 as the original version of this questionnaire was selected. There have since been better questionnaires developed for AIS but even these are not validated for SS. Matching for age and sex had the aim to compare adolescents with similar size and development at the time of surgery. It is also possible that underlying disease progression during follow-up was affecting outcomes.

SS includes a wide range of patients with very different capabilities to assess their quality of life. Some patients, like those with neurofibromatosis, can lead a very normal life, whereas others have severe disabilities and cannot communicate their pain or satisfaction, limiting the reporting of HRQoL.

SRS-24 filled in by caregivers in SS served as a proxy for their quality of life. SS patients can have severe cognitive impairment and some of the patients were not able to answer the questionnaires themselves, which may cause bias. Cognitive impairment was not specifically tested as part of this retrospective cohort study, which may have affected the quality-of-life assessment in children with SS. We feel that the caregivers who know these patients best are the most reliable source of information available regarding HRQoL for these cognitively impaired patients [17].

Even though a significant proportion of the SS group were non-ambulatory, there were no significant differences between SS and AIS patients in the SRS-24 scores either preoperatively or at follow-up. Non-ambulatory SS patients had lower scores only for the general activity domain. The questionnaires reflect how patients evaluate their own health-related quality of life. When patients have lived their whole life as non-ambulatory, they may evaluate themselves as active while if patients with previous ambulatory function lose this function they certainly might evaluate this otherwise [18]. As there were no significant differences in change of HRQoL scores between groups, SS patients and their parents feel that they benefit similarly from spinal fusion compared with AIS.

Although the patient enrollment was ongoing for 14 years, the number of patients in the SS group was quite low. 17 SS patients did not have a preoperative SRS-24 questionnaire available for analyses, significantly reducing the number of patients included. Difference between groups was near the threshold for significance in many domains. It is possible that with a larger study population these differences would become evident and that the ambulatory and healthier SS patients improved the HRQoL outcomes.

### Conclusion

The association of posterior spinal fusion with segmental pedicle screw instrumentation on the HRQoL in SS was comparable to that in AIS. Non-ambulatory status in SS was associated with worse general activity SRS-24 scores but with better improvement in the pain domain. Risk of complications was significantly higher in adolescents with SS than in AIS.

## APPENDIX

**Table 1:** Clinical characteristics and total SRS-24 score for responders and non-responders

Factor	Non-responder (n = 24)	Responder (n = 35)	Difference between groups (CI)
Age at surgery, years ^a^	14.6 (3.7)	14.1 (2.6)	0.6 (–1.2 to 2.4)
Female sex, n (%)	13 (54)	24 (68)	
Major curve, Cobb angle (°)			
Preoperative ^b^	63 [22]	54 [14]	9 (4 to 22)
Postoperative ^b^	15 [12]	15 [9]	1 (–5 to 4)
Correction, % ^b^	80 [20]	70 [20]	5 (–8 to 14)
2-year follow up ^b^	18 [10]	16 [11]	2 (–7 to 7)
Blood loss, mL ^b^	590 [508]	700 [438]	110 (–30 to 330)
Operative time, hours ^b^	3.6 [1.2]	3.7 [1.1]	0.1 (–0.7 to 0.6)
Number of levels fused ^b^	16 [3]	14 [5]	2 (–1 to 4)
Iliac screws/S2AI screws, n	7/4	4/6	
Total SRS-24 score at 2-year follow-up ^b^	4.2 [1.2] ^c^	4.1 [0.4]	0.1 (–0.6 to 0.6)

a and b See Table 2.

c Available for 17/24 patients.

**Table 4:** Clinical characteristics for ambulatory and non-ambulatory SS patients

Factor	Non-ambulatory (n = 10)	Ambulatory (n = 25)	Difference between groups (CI)
Age at surgery, years ^a^	15.1 (2.9)	13.7 (2.5)	1.5 (–0.3 to 3.3)
Female sex, n (%)	6 (60)	18 (51)	
Major curve, Cobb angle (°)			
Preoperative ^b^	59 [25]	51 [11]	8 (–5 to 28)
Postoperative ^b^	16 [9]	13 [8]	3 (–4 to 11)
Correction, % ^b^	70 [10]	70 [20]	0 (–14 to 11)
2-year follow up ^a^	17 (7)	18 (9)	1 (–5 to 6)
Blood loss, mL ^b^	725 [339]	570 [470]	155 (0 to 375)
Operative time, hours ^b^	3.8 [1.3]	3.7 [1.0]	0.1 (–0.4 to 1.3)
Number of levels fused ^b^	17 [1]	13 [3]	4 (3 to 6)

a and b See Table 2.

## Notes

HS: main author, data curation, writing, formal analysis. AS: formal analysis, review and editing. AR: Review and editing. MA: Review and editing. IH: conceptualization, methodology, data curation, supervision, review and editing.

The authors would like to thank Mrs Elisa Wibom-Järvinen for help with the data collection.

Handling co-editors: Paul Gerdhem and Philippe Wagner

*Acta* thanks Engelke Marie Randers and other anonymous reviewers for help with peer review of this manuscript
